# Supplementary material for: Advancements in Research and Treatment Applications of Patient-Derived Tumor Organoids in Colorectal Cancer
Source: Cancers (Basel). 2024 Jul 26;16(15):2671. doi: 10.3390/cancers16152671 (PMC11311786; doi:10.3390/cancers16152671)
Supplement: Supplementary file 1 [file cancers-16-02671-s001.zip › Supplementary Table S1 - Overview of media used.pdf]

**Table S1.** Overview of the various media used.

| | Sato [1] | Vlachogannis [2] | Ganesh [3] | van de Wetering [4]; Ooft [5]; Dijkstra [6] | Cho [7] | Tan [8] | Parikh [9] | Jensen [10] | Chen [11] |
|---|---|---|---|---|---|---|---|---|---|
| Advanced DMEM/F12 | + | + | + | + | - | + | + | + | + |
| N2-containing medium | - | - | | - | + | - | - | - | - |
| R-Spondin conditioned medium | - | - | 20% ** | 20% | 10% | - | 10% | - | - |
| Noggin conditioned medium | - | - | - | 10% | - | - | 10% | - | - |
| Wnt3a conditioned medium | - | | 50%** | - | - | - | 50% | - | - |
| R-Spondin 1 | - | 500 ng/mL | - | - | - | - | - | - | 500 ng/mL |
| Noggin | - | 100 ng/mL | 100 ng/mL ** | - | 100 µg/mL | - | - | - | 100 ng/mL |
| Wnt3a | - | 100 ng/mL | - | - | - | - | - | - | - |
| B27 | 1x | 1x | 1x | 1x | - | 1x | 1x | - | 1x |
| N2 | 1x | 1x | 1x | - | - | 1x | - | - | 1x |
| NAC | 1 mM | - | 1 mM | 1.25 nM | 1.25 nM | 1 mM | 1 mM | - | 1 mM |
| Nicotinamide | - | 4 mM | 10 mM | 10 mM | 10 mM | 5 mM | - | - | - |
| Gastrin | + | 10 nM | 10 nM | 10 nM | 10 nM | 1 nM | 10 nM | - | 10 nM |
| A83-01 | + * | 0.5 µM | 500 nM | 500 nM | 500 nM | 500 nM | 500 nM | - | 500 nM |
| SB202190 | + * | 5 µM | | 3 µM | 3 µM | 5 µM | - | - | 3 µM |
| Y-27632 | + * | 10 µM | 10 µM | - | - | - | 10 µM | 10 µM | - |
| hEGF | + * | 50 ng/mL | 50 ng/mL | 50 ng/mL | 50 ng/mL | - | 50 ng/mL | - | 50 ng/mL |
| Prostaglandine E2 | - | 1 µM | | 10 nM | - | - | - | - | 10 nM |
| bFGF | - | 10 ng/mL | | - | 50 ng/mL | - | 50 ng/mL | - | - |
| FGF10 | - | 10 ng/mL | | - | - | 10 ng/mL | - | - | - |
| Niacinamide | - | - | - | - | - | - | - | - | 10 mM |
| IGF-1 | - | - | - | - | - | - | 100 ng/mL | - | - |
| 2-mercaptoehtanol | - | - | | - | - | - | - | + | - |
| StemProhESC supplement | - | - | | - | - | - | - | + | - |
| Pen-Strep | + | 100 u/mL | | - | - | 1x | 100 U/mL | + | - |
| Primocin | - | - | | 100 µg/mL | - | - | 100 µg/mL | - | - |

| | | | | | | | | | |
|---|---|---|---|---|---|---|---|---|---|
| Normocin | - | - | | - | - | - | - | - | + |
| Gentamicin/amphotericine B | - | - | + | - | - | - | - | 2,5 µg/mL each | + |
| GlutaMAX | 10 mM | 2 mM | 2 mM | - | - | 2 mM | 1x | + | + |
| HEPES | + | - | 10 mM | - | - | 10 mM | 10 nM | - | + |
| Bovine serum albumin | - | 0.01% | | - | - | - | - | + | - |

* Varied; ** These niche factors were removed upon expansion of the organoids. This table is non-exhaustive.

## References


1. Sato, T.; Stange, D.E.; Ferrante, M.; Vries, R.G.; Van Es, J.H.; Van den Brink, S.; Van Houdt, W.J.; Pronk, A.; Van Gorp, J.; Siersema, P.D.; et al. Long-term expansion of epithelial organoids from human colon, adenoma, adenocarcinoma, and Barrett's epithelium. *Gastroenterology* **2011**, *141*, 1762–1772. https://doi.org/10.1053/j.gastro.2011.07.050.
2. Vlachogiannis, G.; Hedayat, S.; Vatsiou, A.; Jamin, Y.; Fernández-Mateos, J.; Khan, K.; Lampis, A.; Eason, K.; Huntingford, I.; Burke, R.; et al. Patient-derived organoids model treatment response of metastatic gastrointestinal cancers. *Science* **2018**, *359*, 920–926. https://doi.org/10.1126/science.aao2774.
3. Ganesh, K.; Wu, C.; O'Rourke, K.P.; Szeglin, B.C.; Zheng, Y.; Sauvé, C.G.; Adileh, M.; Wasserman, I.; Marco, M.R.; Kim, A.S.; et al. A rectal cancer organoid platform to study individual responses to chemoradiation. *Nat. Med.* **2019**, *25*, 1607–1614. https://doi.org/10.1038/s41591-019-0584-2.
4. van de Wetering, M.; Francies, H.E.; Francis, J.M.; Bounova, G.; Iorio, F.; Pronk, A.; van Houdt, W.; van Gorp, J.; Taylor-Weiner, A.; Kester, L.; et al. Prospective derivation of a living organoid biobank of colorectal cancer patients. *Cell* **2015**, *161*, 933–945. https://doi.org/10.1016/j.cell.2015.03.053.
5. Ooft, S.N.; Weeber, F.; Schipper, L.; Dijkstra, K.K.; McLean, C.M.; Kaing, S.; van de Haar, J.; Prevoo, W.; van Werkhoven, E.; Snaebjornsson, P.; et al. Prospective experimental treatment of colorectal cancer patients based on organoid drug responses. *ESMO Open* **2021**, *6*, 100103. https://doi.org/10.1016/j.esmoop.2021.100103.
6. Dijkstra, K.K.; Cattaneo, C.M.; Weeber, F.; Chalabi, M.; van de Haar, J.; Fanchi, L.F.; Slagter, M.; van der Velden, D.L.; Kaing, S.; Kelderman, S.; et al. Generation of Tumor-Reactive T Cells by Co-culture of Peripheral Blood Lymphocytes and Tumor Organoids. *Cell* **2018**, *174*, 1586–1598.e12. https://doi.org/10.1016/j.cell.2018.07.009.
7. Cho, Y.-H.; Ro, E.J.; Yoon, J.-S.; Mizutani, T.; Kang, D.-W.; Park, J.-C.; Kim, T.I.; Clevers, H.; Choi, K.-Y. 5-FU promotes stemness of colorectal cancer via p53-mediated WNT/β-catenin pathway activation. *Nat. Commun.* **2020**, *11*, 5321.
8. Tan, R.; Zhang, Z.; Ding, P.; Liu, Y.; Liu, H.; Lu, M.; Chen, Y.G. A growth factor-reduced culture system for colorectal cancer organoids. *Cancer Lett.* **2024**, *588*, 216737. https://doi.org/10.1016/j.canlet.2024.216737.
9. Parikh, A.Y.; Masi, R.; Gasmi, B.; Hanada, K.I.; Parkhurst, M.; Gartner, J.; Sindiri, S.; Prickett, T.; Robbins, P.; Zacharakis, N.; et al. Using patient-derived tumor organoids from common epithelial cancers to analyze personalized T-cell responses to neoantigens. *Cancer Immunol. Immunother.* **2023**, *72*, 3149–3162. https://doi.org/10.1007/s00262-023-03476-6.
10. Jensen, L.H.; Rogatto, S.R.; Lindebjerg, J.; Havelund, B.; Abildgaard, C.; do Canto, L.M.; Vagn-Hansen, C.; Dam, C.; Rafaelsen, S.; Hansen, T.F. Precision medicine applied to metastatic colorectal cancer using tumor-derived organoids and in-vitro sensitivity testing: A phase 2, single-center, open-label, and non-comparative study. *J. Exp. Clin. Cancer Res.* **2023**, *42*, 115. https://doi.org/10.1186/s13046-023-02683-4.
11. Chen, L.; Tian, B.; Liu, W.; Liang, H.; You, Y.; Liu, W. Molecular Biomarker of Drug Resistance Developed from Patient-Derived Organoids Predicts Survival of Colorectal Cancer Patients. *Front. Oncol.* **2022**, *12*, 855674. https://doi.org/10.3389/fonc.2022.855674.
