# Supplementary material for: Advancements in Research and Treatment Applications of Patient-Derived Tumor Organoids in Colorectal Cancer
Source: Cancers (Basel). 2024 Jul 26;16(15):2671. doi: 10.3390/cancers16152671 (PMC11311786; doi:10.3390/cancers16152671)
Supplement: Supplementary file 1 [file cancers-16-02671-s001.zip › Supplementaty Table S2 - Establisment rate of CRC PDTOs.pdf]

Table S2. Overview of the establishment rates of CRC PDTOs.

First Author	Previous treatment	Source		Success percentage	Comments
		Primary vs metastatic	*Technique*		
Ooft [1]	Both treated and untreated	Metastatic	Biopsy	57% (31/54)	/
Jensen [2]	Yes	Metastatic	Biopsy	54% (44/82)	/
Ganesh [3]	Both treated and untreated	Both	Resection specimens and biopsies	77% (65/84)	Only rectal cancer.
Martini [4]	Both treated and untreated	Both	Resection specimens and biopsies	84% (26/31)	Treatment naïve or first line.
Martini [4]	Yes (≥ 2 lines)	Metastatic (liver)	Biopsies	56% (5/9)	Aggressively pretreated.
Mo [5]	Both treated and untreated	Both	Resection specimens and biopsies	80.6% (58/72) 86.1% (31/36) for CRC PDTOS and 75% (27/36) for liver metastases	In total 36 patients were included, patients could be sampled multiple times.
Bruun [6]	All but one	Metastatic (liver)	Resection	76% (22/29 patients)	However, they used multiple lesions per patient, lesion-wise success rate is 52% (39/75).
Chen [7]	None	Primary	Resection	82% (41/50)	/
Geevimaan [8]		Both	Resection	76% (115/151)	/
Parikh [9]	Unknown	Metastatic	Resection	92% (23/25)	Successful organoid was defined as "proliferation requiring one or more passages"
Xue [10]	Yes (chemoradiotherpy)	Primary	Resection	62.3% (86/138)	/
Pappacio [11]	Both treated and untreated	Both	Biopsies	58.0% (29/50)	Trend toward more successful organoid generation in

					treatment naïve specimens
van de Wetering [12]	No	Primary	Resection	81.5% (22/27)	No rectal cancers included due to neoadjuvant irradiation
Yao [13]	No	Primary	Biopsy	85.7% (96/112)	/
He [14]	Both treated and untreated	Both	Resection	80.8 % (42/52)	/
Weeber [15]	Unknown	Metastatic	Biopsy	71% (10/14)	/

N.B. Non-exhaustive table.

References

1. Ooft, S.N.; Weeber, F.; Schipper, L.; Dijkstra, K.K.; McLean, C.M.; Kaing, S.; van de Haar, J.; Prevoo, W.; van Werkhoven, E.; Snaebjornsson, P.; et al. Prospective experimental treatment of colorectal cancer patients based on organoid drug responses. *ESMO Open* **2021**, *6*, 100103. https://doi.org/10.1016/j.esmoop.2021.100103.
2. Jensen, L.H.; Rogatto, S.R.; Lindebjerg, J.; Havelund, B.; Abildgaard, C.; do Canto, L.M.; Vagn-Hansen, C.; Dam, C.; Rafaelsen, S.; Hansen, T.F. Precision medicine applied to metastatic colorectal cancer using tumor-derived organoids and in-vitro sensitivity testing: A phase 2, single-center, open-label, and non-comparative study. *J. Exp. Clin. Cancer Res.* **2023**, *42*, 115. https://doi.org/10.1186/s13046-023-02683-4.
3. Ganesh, K.; Wu, C.; O'Rourke, K.P.; Szeglin, B.C.; Zheng, Y.; Sauvé, C.G.; Adileh, M.; Wasserman, I.; Marco, M.R.; Kim, A.S.; et al. A rectal cancer organoid platform to study individual responses to chemoradiation. *Nat. Med.* **2019**, *25*, 1607–1614. https://doi.org/10.1038/s41591-019-0584-2.
4. Martini, G.; Belli, V.; Napolitano, S.; Ciaramella, V.; Ciardiello, D.; Belli, A.; Izzo, F.; Avallone, A.; Selvaggi, F.; Menegon Tasselli, F.; et al. Establishment of patient-derived tumor organoids to functionally inform treatment decisions in metastatic colorectal cancer. *ESMO Open* **2023**, *8*, 101198. https://doi.org/10.1016/j.esmoop.2023.101198.
5. Mo, S.; Tang, P.; Luo, W.; Zhang, L.; Li, Y.; Hu, X.; Ma, X.; Chen, Y.; Bao, Y.; He, X.; et al. Patient-Derived Organoids from Colorectal Cancer with Paired Liver Metastasis Reveal Tumor Heterogeneity and Predict Response to Chemotherapy. *Adv. Sci.* **2022**, *9*, e2204097. https://doi.org/10.1002/advs.202204097.
6. Bruun, J.; Kryeziu, K.; Eide, P.W.; Moosavi, S.H.; Eilertsen, I.A.; Langerud, J.; Røsok, B.I.; Totland, M.Z.; Brunsell, T.H.; Pellinen, T.; et al. Patient-Derived Organoids from Multiple Colorectal Cancer Liver Metastases Reveal Moderate Intra-patient Pharmacotranscriptomic Heterogeneity. *Clin. Cancer Res.* **2020**, *26*, 4107-4119.
7. Chen, L.; Tian, B.; Liu, W.; Liang, H.; You, Y.; Liu, W. Molecular Biomarker of Drug Resistance Developed From Patient-Derived Organoids Predicts Survival of Colorectal Cancer Patients. *Front. Oncol.* **2022**, *12*, 855674. https://doi.org/10.3389/fonc.2022.855674.
8. Geevimaan, K.; Guo, J.Y.; Shen, C.N.; Jiang, J.K.; Fann, C.S.J.; Hwang, M.J.; Shui, J.W.; Lin, H.T.; Wang, M.J.; Shih, H.C.; et al. Patient-Derived Organoid Serves as a Platform for Personalized Chemotherapy in Advanced Colorectal Cancer Patients. *Front. Oncol.* **2022**, *12*, 883437. https://doi.org/10.3389/fonc.2022.883437.
9. Parikh, A.Y.; Masi, R.; Gasmi, B.; Hanada, K.I.; Parkhurst, M.; Gartner, J.; Sindiri, S.; Prickett, T.; Robbins, P.; Zacharakis, N.; et al. Using patient-derived tumor organoids from common epithelial cancers to analyze personalized T-cell responses to neoantigens. *Cancer Immunol. Immunother.* **2023**, *72*, 3149–3162. https://doi.org/10.1007/s00262-023-03476-6.
10. Xue, W.; Wang, T.; Yao, J.; Wu, W.; Chen, D.; Yan, B.; Dong, X.; Tang, Y.; Zeng, Y.; He, Y.; et al. Use of patient-derived tumor organoid platform to predict the benefit of postoperative adjuvant chemotherapy for poor responders to neoadjuvant chemoradiotherapy in locally advanced rectal cancer. *Bioeng. Transl. Med.* **2023**, *8*, e10586. https://doi.org/10.1002/btm2.10586.

11. Papaccio, F.; García-Mico, B.; Gimeno-Valiente, F.; Cabeza-Segura, M.; Gambardella, V.; Gutiérrez-Bravo, M.F.; Alfaro-Cervelló, C.; Martinez-Ciarpaglini, C.; Rentero-Garrido, P.; Zúñiga-Trejos, S.; et al. Proteotranscriptomic analysis of advanced colorectal cancer patient derived organoids for drug sensitivity prediction. *J. Exp. Clin. Cancer Res.* **2023**, *42*, 8. https://doi.org/10.1186/s13046-022-02591-z.
12. van de Wetering, M.; Francies, H.E.; Francis, J.M.; Bounova, G.; Iorio, F.; Pronk, A.; van Houdt, W.; van Gorp, J.; Taylor-Weiner, A.; Kester, L.; et al. Prospective derivation of a living organoid biobank of colorectal cancer patients. *Cell* **2015**, *161*, 933–945. https://doi.org/10.1016/j.cell.2015.03.053.
13. Yao, Y.; Xu, X.; Yang, L.; Zhu, J.; Wan, J.; Shen, L.; Xia, F.; Fu, G.; Deng, Y.; Pan, M.; et al. Patient-Derived Organoids Predict Chemoradiation Responses of Locally Advanced Rectal Cancer. *Cell Stem Cell* **2020**, *26*, 17–26.e6. https://doi.org/10.1016/j.stem.2019.10.010.
14. He, X.; Jiang, Y.; Zhang, L.; Li, Y.; Hu, X.; Hua, G.; Cai, S.; Mo, S.; Peng, J. Patient-derived organoids as a platform for drug screening in metastatic colorectal cancer. *Front. Bioeng. Biotechnol.* **2023**, *11*, 1190637.
15. Weeber, F.; van de Wetering, M.; Hoogstraat, M.; Dijkstra, K.K.; Krijgsman, O.; Kuilman, T.; Gadellaa-van Hooijdonk, C.G.; van der Velden, D.L.; Peeper, D.S.; Cuppen, E.P.; et al. Preserved genetic diversity in organoids cultured from biopsies of human colorectal cancer metastases. *Proc. Natl. Acad. Sci. USA* **2015**, *112*, 13308–13311. https://doi.org/10.1073/pnas.1516689112.
